# Cognitive Effects of Combined Aerobic Exercise and Cognitive Training in Older Adults with Mild Cognitive Impairment

**DOI:** 10.1002/alz70858_106044

**Published:** 2025-12-26

**Authors:** Fang Yu, Dereck L. Salisbury, Michael Todd, Lisa S Chow, Anton P. Porsteinsson, Molly Maxfield, Rodney Joseph, Frank LoVecchio, Kathi Heffner, Feng Vankee Lin

**Affiliations:** ^1^ Arizona State University, Phoenix, AZ, USA; ^2^ University of Minnesota, Minneapolis, MN, USA; ^3^ University of Rochester School of Medicine and Dentistry, Rochester, NY, USA; ^4^ Arizona State University Clinical Research Services, Phoenix, AZ, USA; ^5^ University of Rochester Medical Center, Rochester, NY, USA; ^6^ Stanford University, Stanford, CA, USA

## Abstract

**Background:**

Combined Aerobic exercise and Cognitive Training (ACT) may have synergistic effects on cognition, capitalizing on their different mechanisms of action. However, combined physical and cognitive activities showed mixed effects due to widely differing intervention components. To this end, the ACT Trial tested the efficacy of a 6‐month combined cycling and speed of processing (SOP) cognitive training on cognition in community‐dwelling older adults with amnestic mild cognitive impairment.

**Methods:**

The ACT Trial used a 2x2 factorial design to randomize participants equally to four arms: control, cycling only, SOP only, and ACT (cycling+SOP). Executive function and episodic memory were measured with EXAMINER and Brief Visuospatial Memory Test‐Revised (BVMT‐R) at baseline, 3, 6, 12, and 18 months by trained data collectors blinded to group allocation. The mean of z‐transformed scores of EXAMINER and BVMT‐R was used to assess global cognition. Data were analyzed under intention‐to‐treat using linear mixed models with multiple imputed datasets in R.

**Results:**

The sample (*n* = 146; 48% women; 91.8% White) was on average 73.72±5.73 years old, had 16.87±2.88 years of education, and scored 23.45±2.16 on Montreal Cognitive Assessment. All groups showed modest declines in outcomes from 3 months to 18 months (Cohen's ds=0.03–0.04 for executive function; ds=0.18–0.25 for delayed recall; ds=0.07–0.18 for global cognition). Baseline‐adjusted ACT group means on outcomes (0.18 for executive function, 41.7 for memory, and 0.00 for global cognition at 6 months; 0.02, 42.5, 0.20, respectively at 18 months) did not differ significantly from those for cycling only (ps = .19, .56, and .90, respectively at 6 months; *p*s = .66, .81, and .65, respectively at 18 months) or SOP only (ps=.59, .13, and .56, respectively at 6 months; *p*s = .72, .43, and .48, respectively at 18 months). There were 10 possibly study‐related, mild‐to‐moderate adverse events that did not differ between groups.

**Conclusion:**

ACT did not show statistically significant superior effects on cognition relative to its components among older adults with aMCI, although observed values trended in the desired directions. The COVID‐19 pandemic contributed to higher attrition and lower dose delivery, especially in the ACT group, leading to smaller effect sizes than expected.

